# A comparison of policy and direct practice stakeholder perceptions of factors affecting evidence-based practice implementation using concept mapping

**DOI:** 10.1186/1748-5908-6-104

**Published:** 2011-09-07

**Authors:** Amy E Green, Gregory A Aarons

**Affiliations:** 1 Department of Psychiatry, University of California, San Diego, 9500 Gilman Drive (0812), La Jolla, CA, USA 92093-0812; 2 Child and Adolescent Services Research Center at Rady Children's Hospital San Diego, 3020 Children's Way, MC5033, San Diego, CA USA 92123

## Abstract

**Background:**

The goal of this study was to assess potential differences between administrators/policymakers and those involved in direct practice regarding factors believed to be barriers or facilitating factors to evidence-based practice (EBP) implementation in a large public mental health service system in the United States.

**Methods:**

Participants included mental health system county officials, agency directors, program managers, clinical staff, administrative staff, and consumers. As part of concept mapping procedures, brainstorming groups were conducted with each target group to identify specific factors believed to be barriers or facilitating factors to EBP implementation in a large public mental health system. Statements were sorted by similarity and rated by each participant in regard to their perceived importance and changeability. Multidimensional scaling, cluster analysis, descriptive statistics and *t*-tests were used to analyze the data.

**Results:**

A total of 105 statements were distilled into 14 clusters using concept-mapping procedures. Perceptions of importance of factors affecting EBP implementation varied between the two groups, with those involved in direct practice assigning significantly higher ratings to the importance of Clinical Perceptions and the impact of EBP implementation on clinical practice. Consistent with previous studies, financial concerns (costs, funding) were rated among the most important and least likely to change by both groups.

**Conclusions:**

EBP implementation is a complex process, and different stakeholders may hold different opinions regarding the relative importance of the impact of EBP implementation. Implementation efforts must include input from stakeholders at multiple levels to bring divergent and convergent perspectives to light.

## Background

The implementation of evidence-based practices (EBPs) into real-world children's mental health service settings is an important step in improving the quality of services and outcomes for youth and families [1,2]. This holds especially true for clients in the public sector who often have difficulty accessing services and have few alternatives if treatments are not effective. Public mental health services are embedded in local health and human service systems; therefore, input from multiple levels of stakeholders must be considered for effective major change efforts such as implementation of EBP [3,4]. In public mental healthcare, stakeholders include not only the individuals most directly involved--the consumers, clinicians, and administrative staff--but also program managers, agency directors, and local, state, and federal policymakers who may structure organizations and financing in ways more or less conducive to EBPs.

Considerable resources are being used to increase the implementation of EBPs into community care; however, actual implementation requires consideration of multiple stakeholder groups and the different ways they may be impacted. Our conceptual model of EBP implementation in public sector services identifies four phases of implementation--exploration, adoption decision/preparation, active implementation, and sustainment--and notes the importance of considering the interests of multiple levels of stakeholders during each phase to result in positive sustained implementation [5]. Similarly, Grol *et al*. suggest that those implementing innovations such as new guidelines and EBPs in medical settings should consider multiple levels and contexts including the innovation itself, the individual professional, the patient, the social context, the organizational context, and the economic and political context [6]. In order to address such implementation challenges, input from stakeholders representing each level (patient, provider, organization, political) must be considered as part of the overall implementation context.

Stakeholders that view service change from the policy, system, and organizational perspectives may have different views than those from clinical and consumer groups regarding what is important in EBP implementation. For example, at the policy level, bureaucratic structures and processes influence funding and contractual agreements between governmental/funding agencies and provider agencies [7]. Challenges in administering day-to-day operations of clinics, including leadership abilities, high staff turnover, and need for adequate training and clinical supervision may serve as barriers or facilitators to the implementation of EBPs [8]. At the practice level, providers contend with high caseloads, meeting the needs of a variety of clients and their families, and relationships with peers and supervisors [9], while consumers bring their own needs, preferences, and expectations [10]. This characterization, while overly simplified, illustrates how challenges at multiple levels of stakeholders can impact the implementation of EBPs. Some have speculated that one reason why implementation of EBP into everyday practice has not happened is the challenge of satisfying such diverse stakeholder groups that may hold very different values and priorities [11,12]. In order to better identify what factors may be important during implementation, it is essential to understand the perspectives of different stakeholder groups including areas of convergence and divergence.

Efforts to implement EBPs should be guided by knowledge, evidence, and experience regarding effective system, organizational, and service change efforts. Although there is growing interest in identifying key factors likely to affect implementation of EBPs [13-17], much of the existing evidence is from outside the US [18-20] or outside of healthcare settings [21,22]. With regard to implementation of evidence and guidelines in medical settings, systematic reviews have shown that strategies that take into account factors relating to the target group (*e.g*., knowledge and attitudes), to the system (*e.g*., capacity, resources, and service abilities), and to reinforcement from others have the greatest likelihood of facilitating successful implementation [6,23,24].

Additionally, research on implementation of innovations, such as implementing a new EBP, suggests that several major categories of factors may serve as facilitators or barriers to change. For example, changes are more likely to be implemented if they have demonstrated benefits (*e.g*., competitive advantage) [25]. Conversely, higher perceived costs discourage change [25,26]. Change is also more likely to occur and persist if it fits the existing norms and processes of an organization [27-29]. Organizational culture can impact how readily new technologies will be considered and adopted in practice [30], and there is concern that some public sector service organizations may have cultures that are resistant to innovation [3,31]. The presence of supportive resources and leadership also make change much more likely to occur within organizations [32]. On an individual level, change is more likely when individuals believe that implementing a new practice is in their best interest [25,32]. While these studies provide a framework for exploring barriers and facilitating factors to implementation of innovation, most are from settings where factors may be very different than in community-based mental health agencies and public sector services [18,19]. Thus, there are likely to be both common and unique factors in conceptual models from different types of systems and organizations.

While there is generally a dearth of research examining barriers and facilitating factors to implementation of EBPs across multiple service systems, one research team has utilized observation and interview methods to examine barriers and facilitating factors to successful implementation for two specific EBPs in multiple community mental health centers [33,34]. The investigators found three significant common barriers emerged across five implementation sites: deficits in skills and role performance by front-line supervisors, resistance by front-line practitioners, and failure of other agency personnel to adequately fulfill new responsibilities [33]. While barriers such as funding and top level administrative support were common barriers, addressing them was not enough to produce successful implementation, and suggest that a 'synergy' needs to exist involving upper-level administration, program leaders, supervisors, direct services workers, and related professionals in the organization to produce successful EBP implementation in community mental health settings [33]. Additionally, the authors' qualitative findings pointed to a number of facilitating factors for successful implementation across sites, including the use of fidelity monitoring, strong leadership, focused team meetings, mentoring, modeling, and high-quality supervision [34].

Across studies in mental health, medical, and organizational settings, a number of common implementation barriers and facilitating factors occurring at multiple stakeholder levels have been identified. However, despite evidence pointing to the need to consider implementation factors at multiple levels, there is a lack of research examining perspectives of implementation barriers and facilitating factors among those at different stakeholder levels. The overall purpose of the current study is to examine divergent and convergent perspectives towards EBP implementation between those involved in creating and carrying out policy and procedures and those involved in direct practice. Previous research has indicated a need to include multiple perspectives when implementing new programs and policies, but provided few guidelines regarding how to succinctly capture diverse perspectives. The current study uses concept mapping to both assess the level of agreement between policy and direct practice groups with regard to factors important for EBP implementation, and suggests ways to incorporate multiple perspectives into a conceptual framework to facilitate successful implementation.

## Methods

### Study context

The study took place in San Diego County, the sixth most populous county in the United States (at the time of the study). San Diego County is very diverse, comprised of 51% Non-Hispanic Caucasian, 30% Latino, 5% Black, 10% Asian, and 4% other racial/ethnic groups [35]. The county youth mental health system supports over 100 mental health programs. Funding for these programs primarily comes from state allocated mental health dollars provided to and administered by each county. Other sources of funding include public and private insurance. The majority of services are provided through county contracts to community-based organizations, although the county also provides some direct services using their own staff.

### Participants

Participants included 31 stakeholders representing diverse mental health service system organizational levels and a broad range of mental health agencies and programs, including outpatient, day treatment, case management, and residential services. Participants were recruited based on the investigative team's in-depth knowledge of the service system with input from system and organizational participants. First, county children's mental health officials were recruited for participation by the research team. These officials worked with the investigators to identify agency directors and program managers representing a broad range of children and family mental health agencies and programs, including outpatient, day treatment, case management, and residential. There were no exclusion criteria. The investigative team contacted agency directors and program managers by email and/or telephone to describe the study and request their participation. Recruited program managers then identified clinicians, administrative support staff, and consumers for project recruitment. County mental health directors, agency directors, and program managers represent the policy interests of implementation, while clinicians, administrative support staff, and consumers were recruited to represent the direct practice perspectives of EBP implementation. Demographic data including age, race/ethnicity, and gender was collected on all participants. Data on educational background, years working in mental health, and experience implementing EBPs was collected from all participants except consumers.

### Study design

This project used concept mapping, a mixed methods approach with qualitative procedures used to generate data that can then be analyzed using quantitative methods. Concept mapping is a systems method that enables a group to describe its ideas on any topic and represent these ideas visually in a map [36]. The method has been used in a wide range of fields, including health services research and public health [14,37,38].

### Procedure

First, investigators met with a mixed (across levels) group of stakeholder participants and explained that the goal of the project was to identify barriers and facilitators of EBP implementation in public sector child and adolescent mental health settings. They then cited and described three specific examples of EBPs representing the most common types of interventions that might be implemented (*e.g*., individual child-focused (cognitive problem solving skills training), family-focused (functional family therapy), and group-based (aggression replacement training)). In addition to a description of the interventions, participants were provided a written summary of training requirements, intervention duration and frequency, therapist experience/education requirements, cost estimates, and cost/benefit estimates. The investigative team then worked with the study participants to develop the following 'focus statement' to guide the brainstorming sessions: *'*What are the factors that influence the acceptance and use of evidence-based practices in publicly funded mental health programs for families and children?'

Brainstorming sessions were conducted separately with each stakeholder group (county officials, agency directors, program managers, clinicians, administrative staff, and consumers) in order to promote candid response and reduce desirability effects. In response to the focus statement, participants were asked to brainstorm and identify concise statements that described a single concern related to implementing EBP in the youth mental health service system. Participants were also provided with the three examples of EBPs and the associated handouts described above to provide them with easily accessible information about common types of EBPs and their features. Statements were collected from each of the brainstorming sessions, and duplicates statements were eliminated or combined by the investigative team to distill the list into distinct statements. Statements were randomly reordered to minimize priming effects. Researchers met individually with each study participant, gave them a pile of cards representing each distinct statement (one statement per card), and asked each participant to sort similar statements into the same pile, yielding as many piles as the participant deemed appropriate. Finally, each participant was asked to rate each statement describing what influences the acceptance and use of EBPs in publicly funded mental health programs on a 0 to 4 point scale on 'importance' (from 0 'not at all important' to 4 'extremely important') and 'changeability' (from 0 'not at all changeable' to 4 'extremely changeable') based on the questions, 'How important is this factor to the implementation of EBP?' and 'How changeable is this factor?'

### Analysis

Analyses were conducted using concept mapping procedures incorporating multidimensional scaling (MDS) and hierarchical cluster analysis in order to group items and concepts and generate a visual display of how items clustered across all participants. Data from the card sort described above were entered into the Concept Systems software [39], which places the data into a square symmetric similarity matrix [40]. A similarity matrix is created by arranging each participant's card sort data in rows and columns denoting whether or not they placed each pair of statements in the same category. For example, a '1' is placed in row 3, column 1 if someone put statements 1 and 3 in the same pile indicating those cards were judged as similar. Cards not sorted together received a '0.' Matrices for all subjects are then summed yielding an overall square symmetric similarity matrix for the entire sample. Thus, any cell in this matrix can take integer values between 0 and the total number of people who sorted the statements; the value of each cell indicates the number of people who placed each pair in the same pile. The square symmetric similarity matrix is analyzed using MDS to create a two dimensional 'point map,' or a visual representation of each statement and the distance between them based on the square symmetric similarity matrix. Each statement is represented as a numbered point, with points closest together more conceptually similar. The stress value of the point map is a measure of how well the MDS solution maps the original data, indicating good fit. The value should range from 0.10 to 0.35, with lower values indicating a better fit [39]. When the MDS does not fit the original data (*i.e*., the stress value is too high), it means that the distances of statements on the point map are more discrepant from the values in the square symmetrical similarity matrix. When the data maps the solution well, it means that distances on the point map are the same or very similar to those from the square symmetrical similarity matrix.

Cluster analysis is then conducted based on the square symmetric similarity matrix data that was utilized for the MDS analysis in order to delineate clusters of statements that are conceptually similar. An associated cluster map using the grouping of statements is created based on the point map. To determine the final cluster solution, the investigators evaluated potential cluster solutions (*e.g*., 12 clusters, 15 clusters) and then agreed on the final model based on interpretability. Interpretability was determined when consensus was reached among three investigators that creating an additional cluster (*i.e*., going from 14 to 15 cluster groupings) would not increase the meaningfulness of the data. Next, all initial study participants were invited to participate with the research team in defining the meaning of each cluster and identifying an appropriate name for each of the final clusters.

Cluster ratings for 'importance' were computed for both the policy and direct practice groups and displayed on separate cluster rating maps. Additionally, cluster ratings for 'changeability' were computed for both the policy and direct practice groups. Overall cluster ratings, represented by layers on the cluster rating map, are actually a double averaging, representing the average of the mean participant ratings for each statement across all statements in each cluster, so that one value represents each cluster's rating level. Therefore, even seemingly slight differences in averages between clusters are likely to be meaningfully interpretable [41]. *T*-tests were performed to examine differences in mean cluster ratings of both importance and changeability between the policy and direct practice groups, with effect sizes calculated using Cohen's *d *[42].

As part of the concept-mapping procedures, pattern matching was completed to examine the relationships between ratings of importance and ratings of changeability for the policy and direct practice groups. Pattern matching is a bivariate comparison of the cluster average ratings for either multiple types of raters or multiple types of ratings. Pattern matching allows for the quantification of the relationship between two sets of interval level ratings aggregated at the cluster level by providing a Pearson product-moment correlation coefficient, with higher correlations indicating greater congruence. In the current project, we created four pattern matches. First, we conducted one pattern match comparing cluster average ratings on importance between the policy and direct practice groups. Next, we conducted a second analysis comparing cluster average ratings on changeability between the policy and direct practice groups. Finally, pattern matching was used to describe the relationships between cluster importance ratings and cluster changeability ratings for the policy group and the direct practice group.

## Results

### Sample characteristics

The policy group (N = 17) consisted of five county mental health officials, five agency directors, and seven program managers. The direct practice group (N = 14) consisted of six clinicians, three administrative support staff, and five mental health service consumers (*i.e*., parents with children receiving services). The majority of the participants were women (61.3%) and ages ranged from 27 to 60 years, with a mean of 44.4 years (*SD *= 10.9). For the direct practice group, 79% of the sample were female and the average age was 38.07 years (*SD *= 10.8), while the policy group contained only 47% females and had an average age of 49.60 years (*SD *= 8.60). The overall sample was 74.2% Caucasian, 9.7% Hispanic, 3.2% African American, 3.2% Asian American, and 9.7% 'Other.' A majority of participants had earned a Master's degree or higher and almost three-quarters of non-consumer participants had direct experience implementing an EBP. The eight agencies represented in this sample were either operated by or contracted with the county. Agencies ranged in size from 65 to 850 full-time equivalent staff and 9 to 90 programs, with the majority located in an urban setting.

### Statement generation and card sort

Thirteen participants representing all stakeholder types were available to work with the research team in creating the focus statement. Brainstorming sessions with each of the stakeholder groups occurred separately and were approximately one hour in length (*M *= 59.5, *SD *= 16.2). From the brainstorming sessions, a total of 230 statements were generated across the stakeholder groups. By eliminating duplicate statements or combining similar statements, the investigative team then distilled these into 105 distinct statements. The participants sorted the card statements into an average of 11 piles (*M *= 10.7, *SD *= 4.3). The average time it took to sort the statements was 35 minutes, and an additional 25 minutes for statement ratings.

### Cluster map creation

The stress value for the MDS analysis of the card sort data was adequate at 0.26, which falls within the average range of 0.10 and 0.35 for concept-mapping projects. After the MDS analysis determined the point location for statements from the card sort, hierarchical cluster analysis was used to partition the point locations into non-overlapping clusters. Using the concept systems software, a team of three investigators independently examined cluster solutions, and through consensus determined a 14-cluster solution best represented the data.

### Cluster descriptions

Twenty-two of the 31 initial study participants (17 through consensus in a single group meeting and five through individual phone calls) participated with the research team in defining the meaning of each cluster and identifying an appropriate name for each of the 14 final clusters. The clusters included: Clinical Perceptions, Staff Development and Support, Staffing Resources, Agency Compatibility, EBP Limitations, Consumer Concerns, Impact On Clinical Practice, Beneficial Features (of EBP), Consumer Values and Marketing, System Readiness and Compatibility, Research and Outcomes Supporting EBP, Political Dynamics, Funding, and Costs of EBP (statements for each cluster can be found in Additional File 1). In order to provide for broad comparability, we use the overall cluster solution and examine differences in importance and changeability ratings for the policy and practice subgroups. Below, we will describe the general themes presented in each of the fourteen clusters under analysis.

The 'Clinical Perceptions' cluster contains eight statements related to concerns about the role of an EBP therapist, including devaluation, fit with theoretical orientations, and limitations on creativity and flexibility, as well as positive factors such as openness, opportunities to learn skills, and motivations to help clients. The ten statements in the 'Staff Development and Support' cluster represent items thought to facilitate implementation, such as having a staff 'champion' for EBP, having open and adaptable staff who have buy in and are committed to the implementation, and having support and supervision available to clinicians, as well as concerns such as required staff competence levels and abilities to learn EBP skills and staff concerns about evaluations and performance reviews. The three items in the 'Staffing Resources' cluster represent themes relating to competing demands on time, finances, and energy of staff and the challenges of changing staffing structure and requirements needed to implement EBP. The nine items in the 'Agency Compatibility' cluster include themes relating to the fit of EBP with the agency values, structure, requirements, philosophy, and information system, as well as the agencies commitment to education, research, and ensuring fidelity and previous experience implementing EBPs. The 'EBP Limitations' cluster contains three items relating to concerns of EBPs, including how they fit into current models, limitations on the number of clients served, and longer treatment length. The 'Consumer Concerns' cluster contains fourteen items that relate to factors that would encourage EBP use among consumers, such as increased hope for improved results, decreased stigma associated with mental illness when using EBPs, and a fit of the EBP with consumers' culture, comfort, preference, and needs, as well as concerns for consumers, such as expectations for a 'quick fix,' resistance to interventions other than medications, and consumer apprehension about EBPs being seen as 'experiments.' The 'Impact On Clinical Practice' cluster contains eight items related to concerns about how EBP affects the therapeutic relationship, consistency of care, and the ability to individualize treatment plans for clinicians, as well as important characteristics of EBP implementation among clinicians, such as the ability to get a correct diagnosis and the flexibility of EBPs to address multiple client problems and core issues. The 'Beneficial Features (of EBP)' cluster contains three items relating to important features of EBP, including its effectiveness for difficult cases, potential for adaptation without effecting outcomes, and the increased advocacy for its use. The 'Consumer Values and Marketing' cluster contains three items related to the EBP fit with values of consumer involvement and with consumers demand for measureable outcomes, as well as the marketing of EBPs to consumers. The 'System Readiness and Compatibility' cluster contains six items relating to the ability of the service systems to support EBP, including buy in of referral and system partners, as well as the compatibility of EBP with other initiatives being implemented. The 'Research and Outcomes Supporting EBP' cluster contains eleven statements relating to the proven effectiveness and sustainability of EBP service in real work services, as well as the ability of EBPs to measure outcomes for the system. The 'Political Dynamics' cluster contains three items relating to the political fairness in selecting programs, support for implementation of EBPs, and concerns of how multi-sector involvement may work with EBPs. The eight items in the 'Funding' cluster include themes related to the willingness of funding sources to adjust requirements related to productivity, caseloads, and limited time frames to meet the requirements of EBPs, as well as a need for funders to provide clearer contracts and requirements for EBPs. Finally, the 'Costs of EBP' cluster contains nine items relating to concerns regarding the costs of training, equipment, supplies, administrative demands, and hidden costs associated with EBP implementation, as well as strengths of EBPs, such as being billable and providing a competitive advantage for funding. Each of the statements contained in each cluster can be considered a barrier or facilitating factor depending on the manner in which it is addressed. For example, the items related to willingness of funding sources to adjust requirements to fit with the EBP can be considered a barrier to the extent that funding sources fail to adjust to meet the needs of the EBP or a facilitating factor when the funding source is prepared and adjusts accordingly to meet the needs and requirements of EBPs.

### Cluster ratings

**Figure 1:**
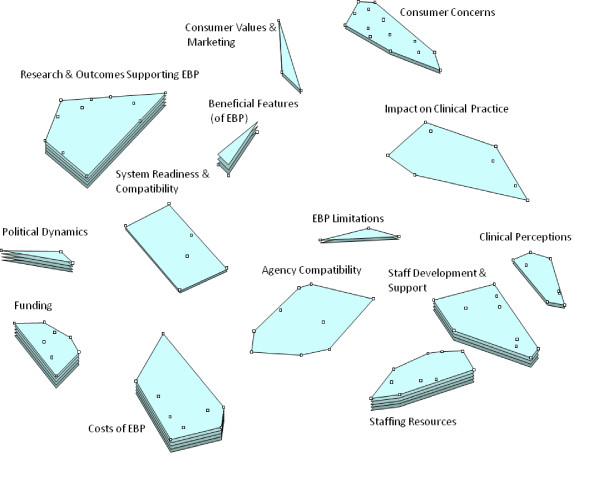
**Policy stakeholder importance cluster rating map**.

**Figure 2:**
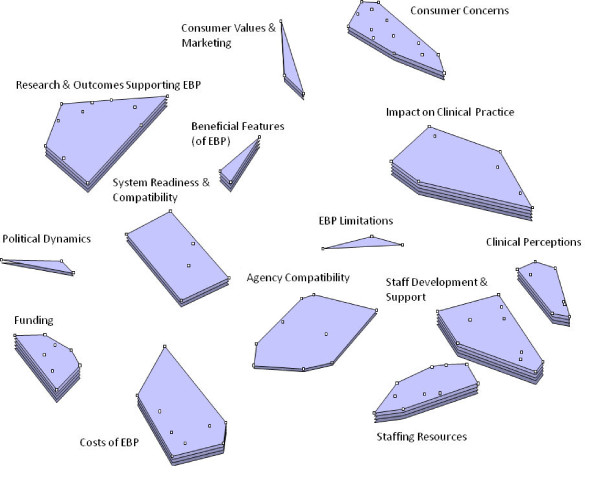
**Practice stakeholder importance cluster rating map**.

Figures 1 and 2 show the cluster rating maps for barriers and facilitators of EBP implementation separately for the policy group and practice group participants. In each figure, the number of layers in each cluster's stack indicates the relative level of importance participants ascribed to factors within that cluster. A smaller cluster indicates that statements were more frequently sorted into the same piles by participants (indicating a higher degree of similarity). Proximity of clusters to each other indicates that clusters are more related to nearby clusters than clusters further away. Overall orientation of the cluster-rating map (*e.g*., top, bottom, right, or left) does not have any inherent meaning.

Tables 1 and 2 present the mean policy and practice group ratings for each cluster, the ranking order of the cluster, and the related *t*-test and Cohen's effect size (*d*) statistics for perceived importance and changeability (respectively). Only two clusters of the fourteen clusters were rated significantly different from each other in importance between the two groups. These significant differences occurred on the 'Impact On Clinical Practice' (*d *= 1.33) and 'Clinical Perceptions' (*d *= 0.69) clusters, where the direct practice group rated the clusters as significantly more important than those in groups that had oversight of policies and procedures. Additionally, *t*-tests of mean differences among the 14 clusters only indicated significant differences in changeability ratings between the groups for financial factors, with the policy group rating them significantly less changeable.

**Table 1:** Mean differences in importance ratings for policy and practice groups

	Policy (N = 17)			Practice (N = 14)			T-test; p-value	ES *Cohen*
	*Rank*	*M*	*SD*	*Rank*	*M*	*SD*		*d*
Staffing resources	1	3.21	0.33	5	3.11	0.44	*t *= 0.76, *p *= 0.45	0.27
Costs of EBP	2	3.13	0.53	4	3.14	0.72	*t *= 0.02, *p *= 0.99	0.01
Funding	3	3.11	0.35	2	3.24	0.81	*t *= 0.60, *p *= 0.55	0.21
Research and Outcomes Supporting EBP	4	3.09	0.44	6	3.09	0.60	*t *= 0.00, *p *= 0.99	0.00
Staff Development and Support	5	3.06	0.28	1	3.28	0.49	*t *= 1.56, *p *= 0.13	0.55
Political Dynamics	6	2.92	0.60	12	2.88	0.78	*t *= 0.16, *p *= 0.87	0.06
Beneficial features (of EBP)	7	2.82	0.47	8	3.07	0.75	*t *= 1.12, *p *= 0.27	0.39
Consumer Values and Marketing	8	2.72	0.47	9	3.05	0.69	*t *= 1.54, *p *= 0.14	0.54
Consumer Concerns	9	2.71	0.37	10	3.01	0.66	*t *= 1.58, *p *= 0.13	0.55
Clinical Perceptions	10	2.70	0.63	6	3.09	0.45	*t *= 2.63, *p *= 0.01	0.69
EBP Limitations	11	2.67	0.65	14	2.74	0.69	*t *= 0.30, *p *= 0.77	0.10
System Readiness and Compatibility	11	2.67	0.57	11	2.96	0.71	*t *= 1.21, *p *= 0.24	0.43
Agency Compatibility	13	2.52	0.47	13	2.87	0.58	*t *= 1.90, *p *= 0.07	0.67
Impact on Clinical Practice	14	2.48	0.50	3	3.21	0.59	*t *= 3.72, *p *< 0.01	1.33

**Table 2:** Mean differences in changeability ratings for policy and practice groups

	Policy (N = 17)			Practice (N = 14)			T-test; p-value	ES *Cohen*
	*Rank*	*M*	*SD*	*Rank*	*M*	*SD*		*d*
Clinical Perceptions	1	2.69	0.63	1	2.71	0.57	*t *= 0.11, *p *= 0.92	0.04
Staff Development and Support	2	2.57	0.35	4	2.51	0.62	*t *= 0.74, *p *= 0.47	0.11
Consumer Values and Marketing	3	2.55	0.55	3	2.57	0.62	*t *= 0.11, *p *= 0.92	0.04
Impact on Clinical Practice	4	2.43	0.60	2	2.69	0.75	*t *= 1.02, *p *= 0.32	0.37
Consumer Concerns	5	2.42	0.56	6	2.49	0.73	*t *= 0.32, *p *= 0.75	0.11
Research and Outcomes supporting EBP	6	2.30	0.44	4	2.51	0.55	*t *= 1.20, *p *= 0.24	0.42
Agency Compatibility	7	2.24	0.54	8	2.41	0.62	*t *= 0.80, *p *= 0.43	0.29
Beneficial Features (of EBP)	7	2.24	0.44	14	2.24	0.80	*t *= 0.01, *p *= 0.99	0.00
Staffing Resources	9	2.17	0.49	10	2.39	0.61	*t *= 1.09, *p *= 0.29	0.39
System Readiness and Compatibility	10	2.15	0.48	6	2.49	0.60	*t *= 1.75, *p *= 0.09	0.61
Political Dynamics	11	2.10	0.74	12	2.36	0.90	*t *= 0.88, *p *= 0.39	0.31
EBP Limitations	12	2.04	0.68	11	2.38	0.74	*t *= 1.34, *p *= 0.19	0.47
Costs of EBP	13	1.97	0.47	9	2.40	0.70	*t *= 1.94, *p *= 0.07	0.71
Funding	14	1.69	0.47	13	2.26	0.97	*t *= 2.14, *p *= 0.04	0.75

### Pattern matching

Pattern matching was used to examine bivariate comparison of the cluster average ratings. Peasons's product moment correlation coefficients indicate the degree to which the groups converge or diverge in perceptions of importance and changeability. In general, agreement between the two groups regarding the cluster importance ratings (*r *= 0.44) was evident. When ranking in order or importance ratings, five of the highest ranked six clusters were rated similarly in importance for the two groups (Funding, Costs of EBP, Staffing Resources, Research and Outcomes Supporting EBP, and Staff Development and Support). There was also concordance between the least important factors with System Readiness and Compatibility, Agency Compatibility, and Limitations of EBP all falling in the bottom four rankings for both groups. Results from the pattern matching of changeability ratings revealed few differences between the two groups for the 14 domains as indicated by the high between-groups correlation (*r *= 0.78). Clinical Perceptions were rated most amenable to change in both the policy (*M *= 2.69) and practice groups (*M *= 2.71).

Pattern matching was also used to describe the discrepancies between cluster importance ratings and cluster changeability ratings for both the policy and practice groups. There was a small positive correlation between importance ratings and changeability ratings for those involved in direct practice (*r *= 0.20) where high importance ratings were associated with higher changeability ratings. Conversely, there was a negative correlation between importance and changeability for the policy group (*r *= -0.39) whereby those factors rated as most important were less likely to be rated as amendable to change. Resource issues emerged in two distinct dimensions: financial (Funding, Costs of EBPs) and human (Staffing Resources, Staff Development and Support), which were both rated among the highest levels of importance for both groups. Financial domains (Funding and Costs) were rated among the least amendable to change by both groups; however, Staff Development and Support was rated as more changeable by both groups.

## Discussion

The current study builds on our previous research in which we identified multiple factors likely to facilitate or be barriers to EBP implementation in public mental health services [14]. In the present study, we extended findings to assess differences in policy and practice stakeholder perspectives on what it takes to implement EBP. These include concerns about the strength of the evidence base, how agencies with very limited financial and human resources can bear the costs attendant to changing therapeutic modalities, concerns about effects on clinical practice, consumer concerns about quality and stigma, and potential burden for new types of services. Each cluster or factor can be considered a facilitator or barrier to EBP implementation to the degree that the issues are effectively addressed. For example, funding is a facilitator when sufficient to support training, infrastructure, and fidelity monitoring, but would be considered a barrier if not sufficient to meet these and other common issues for EBP implementation.

While there was a great deal of agreement between administrators/policymakers and those involved in direct practice in regard to the most important and least important barriers and facilitating factors, there were also differences. In regard to areas of agreement, these results can be used target and address areas of concern prior to implementation. For example, resource availability (financial and staffing) appeared to be especially salient for both those at the policy and practice levels. Such services can be under-funded and often contend with high staff turnover often averaging 25% per year or more [43]. Funds for mental health and social services may face competing priorities of legislatures that may favor funding to cover other increasing costs such as Medicaid and prisons [44]. Such concerns must not be overlooked when implementing EBPs in public sector settings, and may be addressed by higher policy level initiatives that have the power to change factors that appear unalterable at the agency and practitioner levels. Hence, we suggest that it is necessary for both policy makers and those involved in direct practice to be consulted and involved in a collaborative way when designing strategies to implement EBPs.

Conversely, contrasting stakeholder group perceptions suggests that taking different perspectives into account can inform implementation process and potentially outcomes, because satisfying the needs of multiple stakeholders has been cited as one of the major barriers to successful implementation of EBPs [11,12]. Differences across stakeholder groups in their perceptions of the importance and changeability of factors affecting EBPs point to the need for increased communication among stakeholders to help develop a more complete understanding of what affects implementation. Tailoring content and delivery method of EBP and related implementation information for particular stakeholders may promote more positive attitudes toward implementation of change in service models. For example, highlighting positive personal experiences along with research results of EBP on clinical practice may be an effective strategy for practitioners, administrative staff, and consumers; however, policy makers may be more swayed by presentations of long-term cost effectiveness data for EBPs.

Additionally, a better understanding of different stakeholder perspectives may lead to better collaboration among different levels of stakeholders to improve services and service delivery. Too often, processes are less than collaborative due to time pressures, meeting the demands of funders (*e.g*., federal, state), and the day-to-day work of providing mental health services. Processes for such egalitarian multiple stakeholders input can facilitate exchange between cultures of research and practice [45].

The work presented here also adds to the knowledge base and informs our developing conceptual model of implementation. This study fits with our conceptual model of implementation that acknowledges the importance of considering system, organizational, and individual levels and the interests of multiple stakeholders during the four phases of implementation (exploration, adoption/preparation, active implementation, sustainment) [5]. The model notes that different priorities might be more or less relevant for different groups, and that if a collaborative process in which multiple stakeholder needs are addressed is employed, implementation decisions and planning will be more likely to result in positive implementation outcomes [46].

While this study was conducted in a public mental health system, it is important to note that there are numerous commonalities across public service sectors that increase the likely generalizability of the findings presented here [5]. For example, mental health, child welfare, and alcohol/drug service settings commonly operate with a central authority that sets policy and directs funding mechanisms such as requests for proposals and contracts for services. Depending on the context, these directives may emanate from state or county level government agencies or some combination of both (*e.g*., state provides directives or regulations for county level use of funds or services). In addition, mental health managers, clinicians, and consumers may also be involved with child welfare and/or alcohol/drug services under contracts or memorandums of understanding with agencies or organizations in other sectors. Indeed, it is not uncommon for consumers to be involved in services in more than one sector [47]

### Limitations

Some limitations of the present study should be noted. First, the sample was derived from one public mental health system which may limit generalizability. Hence, different participants could have generated different statements and rated them differently in terms of their importance and changeability. However, San Diego is among the six most populous counties in the United States and has a high degree of racial and ethnic diversity. Thus, while not necessarily generalizable to all other settings, the present findings likely represent many issues and concerns that are at play in other service settings. Additionally, the sample size poses a limitation as we were unable to assess differences between specific stakeholder types (*i.e*., county officials versus program managers) because there is insufficient power. By grouping participants into policy/administrators and those in direct practice, we were able to create group sizes large enough to detect medium to large effect sizes. It would not have been feasible to recruit samples for each of six stakeholder groups large enough to find significant differences using concept mapping procedures. We opted to include a greater array of stakeholder types at the cost of larger stakeholder groups. Future studies may consider examining larger numbers of fewer stakeholder types (*i.e*., only county officials, program directors, and clinicians) to make comparisons among specific groups. Another limitation concerns self-report nature of the data collected, because some have suggested that the identification of perceived barriers by practitioners are often part of a 'sense-making' strategy that may have varied meanings in different organizational contexts and may not relate directly to actual practice [48,49]. However, in the current study, the focus statement was structured so that participants would express their beliefs based on general experiences with EBP rather than one particular project, hence reducing the likelihood of *post hoc *sense making and increasing the generalizability. It should also be noted that participants could have identified different statements and rated them differently for specific EBP interventions.

## Conclusions

Large (and small, for that matter) implementation efforts require a great deal of forethought and planning in addition to having appropriate structures and processes to support ongoing instantiation and sustainment of EBPs in complex service systems. The findings from this study and our previous work [5,14] provide a lens through which implementation can be viewed. There are other models and approaches to be considered, which may be less or more comprehensive than the one presented here [15-17]. Our main message is that careful consideration of factors at multiple levels and of importance to multiple stakeholders should be explored, understood, and valued as part of the collaborative implementation process through the four implementation phases of exploration, adoption decision/planning, active implementation, and sustainment [5].

There are many 'cultures' to be considered in EBP implementation. These include the cultures of government, policy, organization management, clinical services, and consumer needs and values. In order to be successful, the implementation process must acknowledge and value the needs, exigencies, and values present and active across these strata. Such cultural exchange as described by Palinkas *et al. *[45] will go a long way toward improving EBP implementation process and outcomes.

## Competing interests

Gregory A. Aarons is an Associate Editor of *Implementation Science*. The authors declare that they have no competing interests.

## Authors' contributions

GA contributed to the theoretical background and conceptualization of the study, collected the original data, and supervised the analyses in the current project. AG contributed to the theoretical background and conceptualization of the study and conducted the related analyses. Both GA and AG contributed to the drafting of this manuscript and approved the final manuscript.

## Supplementary Material

Additional file 1 **Concept mapping statements by cluster**. List of each of the statements and their corresponding clusters created as a result of the concept mapping procedure. Click here for file

## References

[B1] Hoagwood K Olin S The NIMH blueprint for change report: Research priorities in child and adolescent mental health J Am Acad Child Adolesc Psychiatry 2002 41 760 767 10.1097/00004583-200207000-00006 12108799

[B2] Jensen PS Commentary: The next generation is overdue J Am Acad Child Adolesc Psychiatry 2003 42 527 530 10.1097/01.CHI.0000046837.90931.A0 12707556

[B3] Backer TE David SL Soucy GE Reviewing the Behavioral Science Knowledge Base on Technology Transfer (NIDA Research Monograph 155, NIH Publication No. 95-4035) 1995 Rockville, MD: National Institute on Drug Abuse 8594449

[B4] Ferlie EB Shortell SM Improving the quality of health care in the United Kingdom and the United States: a framework for change Milbank Q 2001 79 281 315 10.1111/1468-0009.00206 11439467 PMC2751188

[B5] Aarons GA Hurlburt M Horwitz S Advancing a conceptual model of evidence-based practice implementation in public service sectors Adm Policy Ment Health 2011 38 4 23 10.1007/s10488-010-0327-7 21197565 PMC3025110

[B6] Grol R Wensing M What drives change? Barriers to and incentives for achieving evidence-based practice Med J Aust 2004 180 S57 S60 15012583 10.5694/j.1326-5377.2004.tb05948.x

[B7] Glisson C Hasenfeld Y Structure and technology in human service organizations Human services as complex organizations 1992 Thousand Oaks, CA: Sage Publications 184 202

[B8] Lehman WEK Greener JM Simpson DD Assessing organizational readiness for change J Subst Abuse Treat 2002 22 197 209 10.1016/S0740-5472(02)00233-7 12072164

[B9] Aarons GA Mental health provider attitudes toward adoption of evidence-based practice: The Evidence-Based Practice Attitude Scale (EBPAS) Adm Policy Ment Health 2004 6 61 74 10.1023/b:mhsr.0000024351.12294.65 PMC1564126 15224451

[B10] Laing A Hogg G Political exhortation, patient expectation and professional execution: Perspectives on the consumerization of health care Brit J Manage 2002 13 173 188 10.1111/1467-8551.00230

[B11] Hermann RC Chan JA Zazzali JL Lerner D Aligning measurement-based quality improvement with implementation of evidence-based practices Adm Policy Ment Health 2006 33 636 645 10.1007/s10488-006-0055-1 16775755

[B12] Innvaer S Vist G Trommald M Oxman A Health policy-makers' perceptions of their use of evidence: a systematic review J Health Serv Res Policy 2002 7 239 244 10.1258/135581902320432778 12425783

[B13] Aarons GA Measuring provider attitudes toward evidence-based practice: Consideration of organizational context and individual differences Child Adolesc Psychiatr Clin N Am 2005 14 255 271 10.1016/j.chc.2004.04.008 15694785 PMC1564127

[B14] Aarons GA Wells RS Zagursky K Fettes DL Palinkas LA Implementing evidence-based practice in community mental health agencies: A multiple stakeholder analysis Am J Public Health 2009 99 2087 2095 10.2105/AJPH.2009.161711 19762654 PMC2759812

[B15] Damschroder L Aron D Keith R Kirsh S Alexander J Lowery J Fostering implementation of health services research findings into practice: A consolidated framework for advancing implementation science Implement Sci 2009 4 50 10.1186/1748-5908-4-50 19664226 PMC2736161

[B16] Fixsen DL Naoom SF Blase KA Friedman RM Wallace F Implementation Research: A synthesis of the literature 2005 Tampa, FL: University of South Florida, Louis de la Parte Florida Mental Health Institute, The National Implementation Research Network (FMHI Publication #231)

[B17] Greenhalgh T Robert G Macfarlane F Bate P Kyriakidou O Diffusion of innovations in service organizations: Systematic review and recommendations Milbank Q 2004 82 581 629 10.1111/j.0887-378X.2004.00325.x 15595944 PMC2690184

[B18] Dopson S Fitzgerald L The role of the middle manager in the implementation of evidence-based health care J Nurs Manag 2006 14 43 51 10.1111/j.1365-2934.2005.00612.x 16359445

[B19] Dopson S Locock L Gabbay J Ferlie E Fitzgerald L Evidence-based medicine and the implementation gap Health: An Interdisciplinary Journal for the Social Study of Health, Illness and Medicine 2003 7 311 330 10.1177/1363459303007003004

[B20] Morgenstern J Effective technology transfer in alcoholism treatment Subst Use Misuse 2000 35 1659 1678 10.3109/10826080009148236 11138703

[B21] Frambach RT Schillewaert N Organizational innovation adoption: A multi-level framework of determinants and opportunities for future research J Bus Res Special Issue: Marketing theory in the next millennium 2002 55 163 176

[B22] Klein KJ Conn AB Sorra JS Implementing computerized technology: An organizational analysis J Appl Psychol 2001 86 811 824 11596799 10.1037/0021-9010.86.5.811

[B23] Davis DA Thomson MA Oxman AD Haynes RB Changing physician performance: a systematic review of the effect of continuing medical education strategies JAMA 1995 274 700 10.1001/jama.274.9.700 7650822

[B24] Solomon DH Hashimoto H Daltroy L Liang MH Techniques to improve physicians' use of diagnostic tests: a new conceptual framework JAMA 1998 280 2020 10.1001/jama.280.23.2020 9863854

[B25] Buchanan D Fitzgerald L Ketley D Gollop R Jones JL Lamont SS Neath A Whitby E No going back: A review of the literature on sustaining organizational change Int J of Manage Rev 2005 7 189 205 10.1111/j.1468-2370.2005.00111.x

[B26] Rimmer M Macneil J Chenhall R Langfield-Smith K Watts L Reinventing Competitiveness: Achieving Best Practice in Australia 1996 South Melbourne, Australia: Pitman

[B27] Kotter JP Leading Change: Why Transformation Efforts Fail. (Cover story) Harvard Bus Rev 1995 73 59 67

[B28] Lozeau D Langley A Denis J-L The corruption of managerial techniques by organizations Human Relations 2002 55 537 564 10.1177/0018726702055005427

[B29] Pettigrew AM Ferlie E McKee L Shaping strategic change: Making change in large organizations: The case of the National Health Service 1992 London: Sage Publications

[B30] Hemmelgarn AL Glisson C Dukes D Emergency room culture and the emotional support component of Family-Centered Care Child Health Care 2001 30 93 110 10.1207/S15326888CHC3002_2

[B31] Diamond MA Innovation and diffusion of technology: A human process Consult Psychol J 1996 48 221 229

[B32] Dale BG Boaden RJ Wilcox M McQuater RE Sustaining continuous improvement: What are the key issues? Quality Engineering 1999 11 369 377 10.1080/08982119908919253

[B33] Rapp CA Etzel-Wise D Marty D Coffman M Carlson L Asher D Callaghan J Holter M Barriers to evidence-based practice implementation: results of a qualitative study Community Ment Health J 2010 46 112 118 10.1007/s10597-009-9238-z 19685185

[B34] Rapp CA Etzel-Wise D Marty D Coffman M Carlson L Asher D Callaghan J Whitley R Evidence based practice implementation strategies: Results of a qualitative study Community Ment Health J 2008 44 213 224 10.1007/s10597-007-9109-4 17973191

[B35] US Census Bureau State and county quick facts: San Diego County, California 2010 US Census Bureau

[B36] Trochim WM An introduction to concept mapping for planning and evaluation Eval Program Plann 1989 12 1 16 10.1016/0149-7189(89)90016-5

[B37] Burke JG O'Campo P Peak GL Gielen AC McDonnell KA Trochim WM An introduction to concept mapping as a participatory public health research methodology Qual Health Res 2005 15 1392 1410 10.1177/1049732305278876 16263919

[B38] Trochim WM Cook JA Setze RJ Using concept mapping to develop a conceptual framework of staff's views of a supported employment program for individuals with severe mental illness J Consult Clin Psychol 1994 62 766 775 7962880 10.1037//0022-006x.62.4.766

[B39] Concept Systems The Concept System software^®^ Ithaca, NY 2007 4.147

[B40] Kane M Trochim W Concept mapping for planning and evaluation 2007 Thousand Oaks, CA: Sage Publications, Inc

[B41] Trochim WMK Stillman FA Clark PI Schmitt CL Development of a model of the tobacco industry's interference with tobacco control programmes Tob Control 2003 12 140 147 10.1136/tc.12.2.140 12773723 PMC1747710

[B42] Cohen J Statistical power analysis for the behavioral sciences 1988 2 Hillsdale, NJ: Erlbaum

[B43] Aarons G Sawitzky A Organizational climate partially mediates the effect of culture on work attitudes and staff turnover in mental health services Adm Policy Ment Health 2006 33 289 301 10.1007/s10488-006-0039-1 16544205 PMC1564125

[B44] Domino ME Norton EC Morrissey JP Thakur N Cost Shifting to Jails after a Change to Managed Mental Health Care Health Serv Res 2004 39 1379 1402 10.1111/j.1475-6773.2004.00295.x 15333114 PMC1361075

[B45] Palinkas LA Aarons GA Chorpita BF Hoagwood K Landsverk J Weisz JR Cultural exchange and implementation of evidence-based practices Res Social Work Prac 2009 19 602 612 10.1177/1049731509335529

[B46] Proctor E Silmere H Raghavan R Hovmand P Aarons G Bunger A Griffey R Hensley M Outcomes for implementation research: Conceptual distinctions, measurement challenges, and research questions Adm Policy Ment Health 2011 38 65 76 10.1007/s10488-010-0319-7 20957426 PMC3068522

[B47] Aarons GA Brown SA Hough RL Garland AF Wood PA Prevalence of adolescent substance use disorders across five sectors of care J Am Acad Child Adolesc Psychiatry 2001 40 419 426 10.1097/00004583-200104000-00010 11314567

[B48] Checkland K Harrison S Marshall M Is the metaphor of'barriers to change'useful in understanding implementation? Evidence from general medical practice J Health Serv Res Policy 2007 12 95 10.1258/135581907780279657 17407659

[B49] Checkland K Coleman A Harrison S Hiroeh U 'We can't get anything done because...': making sense of'barriers' to Practice-based Commissioning J Health Serv Res Policy 2009 14 20 10.1258/jhsrp.2008.008043 19103913

