# Supplementary material for: A comparison of policy and direct practice stakeholder perceptions of factors affecting evidence-based practice implementation using concept mapping
Source: Implement Sci. 2011 Sep 7;6:104. doi: 10.1186/1748-5908-6-104 (PMC3178500; doi:10.1186/1748-5908-6-104)
Supplement: Additional file 1 — Concept mapping statements by cluster. List of each of the statements and their corresponding clusters created as a result of the concept mapping procedure. [file 1748-5908-6-104-S1.DOC]

**Additional File 1.** Concept Mapping Statements by Cluster

**Cluster 1. Clinical Perceptions**

1 perceived devaluation of the therapist's role

14 EBP provides new skills and clinical perspectives

29 staff desire/motivation for better client outcomes

34 clinician knowledge and perceptions of EBP

35 fit between therapist and the EBP (e.g. theoretical orientation, preference for individual vs. family,

group, or systems therapy)

48 EBP challenges provider professional relationships and status (e.g., was experienced but now having to be beginner)

71 interest, openness, and curiosity of clinicians and managers

82 perception that EBP limits clinician creativity and flexibility

**Cluster 2. Staff Development & Support**

9 staff "champion" or "local opinion leader" for EBP

10 EBP's potential to reduce staff burnout

11 required competence/qualifications of providers

19 openness and adaptability of staff, clinicians, and managers

21 buy-in and commitment of staff and trainees

75 training can be used for clinician licensure hours and continuing education credits

77 staff concern about how they will be evaluated on use of EBP (e.g., performance review)

90 staff abilities and potential to develop required EBP skills

102 level of support and supervision for clinicians

104 impact on clinician productivity requirements

**Cluster 3. Staffing Resources**

2 challenge of changing existing staffing structure (e.g., group or individual treatment)

4 administrative demands of EBP for clinicians (e.g., record keeping)

7 loss of clinicians from general program staff after EBP training

12 staff turnover

16 time for training, supervision and ongoing training in EBP model

25 method of roll-out, (e.g., having only one clinician per agency trained in an EBP)

46 enough available staff to implement EBP

65 competing demands for staff time and energy

69 training of current staff to be EBP trainers

81 time and resources available for supervision and training

**Cluster 4. Agency Compatibility**

54 heterogeneity or preference within each organization

6 EBP compatibility with agency values, philosophy, and vision

40 program commitment to education and research

88 agency's previous experience implementing an EBP

36 logistics of EBP [e.g. location (clinic, school, home-based), transportation, scheduling]

41 new or altered forms or documentation requirements (e.g. measures, clinical forms)

96 ongoing agency commitment to ensure fidelity

93 EBP fit with existing information system (computer)

8 graduate school willingness to incorporate teaching new treatment models and practices

**Cluster 5. EBP Limitations**

94 incorporating a structured practice into a current model

55 limited number of clients that can be served with an EBP

67 EBP takes longer than typical treatment or patient stay

**Cluster 6. Consumer Concerns**

20 EBP's increased demands on consumers (e.g., more meetings, outcome measures)

26 fit of EBP with consumers’ culture

51 consumers' increased hope for improved results from the EBP

52 consumer trust in the mental health system

56 consumer concern about inappropriate use of medication as quick fix

57 EBP decreases stigma of having a mental health problem and seeking treatment

64 child and parent expectation for an external "quick fix"

66 consumer apprehension about risk of EBP and being seen as "experiments"

68 consumer and family engagement with program or EBP

76 openness of clients and families to try new treatment models

80 consumer comfort and satisfaction with the EBP

84 consumer resistance to interventions other than medications

99 youth vs. caregiver treatment preference (e.g., use of evidence-based medication)

103 EBP fit with consumer needs and other family demands

**Cluster 7.**  **Impact on Clinical Practice**

22 EBP effect on consistency of care

23 ability to get correct diagnosis

28 skepticism that EBPs are just the "latest trend"

43 ability to individualize treatment plans

44 flexibility of the EBP to address multiple client problems

70 the EBP addresses core/underlying issues vs. symptoms and behaviors

91 EBP implementation effect on quality of therapeutic relationship

95 confusion about what an EBP is

**Cluster 8.**  **Beneficial Features (of EBP)**

3 EBP seen as effective for difficult cases

61 increased advocacy for use EBPs

86 potential for adaptation of the EBP without affecting outcomes

**Cluster 9.**  **Consumer Values & Marketing**

13 empowered consumers demanding measurable outcomes

18 EBP fit with system of care values (e.g., family involvement)

72 communicating and marketing EBP to consumers

**Cluster 10. System Readiness & Compatibility**

31 education, training, and buy-in of referral partners

38 meeting standards for accountability and effective services

50 possible liability for not using an EBP if it has superior outcomes

60 EBP buy-in and support from other system partners (e.g., schools, juvenile justice, alcohol and drugs, etc.)

78 EBP compatibility with other initiatives that are being implemented

97 incentives for successful implementation of EBP

**Cluster 11. Research & Outcomes Supporting EBP**

27 fidelity and outcomes of EBP support sustainability of services

30 EBP proven effective in real-world settings

33 EBP measures meaningful outcomes for the system

49 ongoing effectiveness, proof that the EBP is still working

53 the EBP is responsive to new research/evidence

73 EBP more likely to use data to show client progress

74 EBP supports other system goals (cross-sector)

79 validity and reliability of evidence

83 generalizability of EBPs research/evidence to other groups (different cultures, treatment settings, diagnostic groups)

92 knowledge of results of EBP in other localities

100 the EBP has specific targeted outcome goals

**Cluster 12. Political Dynamics**

5 multi-sector involvement may hinder delivery of an EBP

59 political/administrative support for the EBP

89 county/government responsibility for fairness in selecting programs to implement EBP

**Cluster 13. Funding**

15 willingness of funding sources to adjust requirements (productivity, case-load, time-frames)

37 EBP match with goals of local funding sources

42 cost savings across other systems

45 EBP fit with insurance limits/options

47 lower long-term cost if future need for treatment is reduced

85 available funding for EBP

87 funders provide clear terms/contracts and auditing requirements for EBPs

105 lower cost per client after start-up costs

**Cluster 14.**  **Costs of EBP**

17 other agency/program readiness to support efforts with referrals, staff time, funds, and services

24 costs for equipment, fidelity measures, and specialized supervision

32 cost of training

39 cost of increased administrative demands

58 having clear knowledge of the exact costs (hidden costs; e.g. specific outcome measures,

retraining, etc.)

62 EBP and related tasks are billable

63 potential benefit for agency/program (revenues, competitive advantage)

98 cost of obtaining or reconfiguring space for EBP

101 potential risk for agency (cost-benefit in regard to outcomes)
